# Mitochondrial numbers increase during glucose deprivation in the slime mold *Physarum polycephalum*

**DOI:** 10.1007/s00709-019-01410-1

**Published:** 2019-07-02

**Authors:** Christina Oettmeier, Hans-Günther Döbereiner

**Affiliations:** 1 Institut für Biophysik, Universität Bremen, NW1 Raum N4260, Otto-Hahn-Allee 1, 28359 Bremen, Germany; 2 Institut für Biophysik, Universität Bremen, NW1 Raum O4040, Postfach 330440, 28334 Bremen, Germany

**Keywords:** *Physarum polycephalum*, Mitochondria, Stereology, Glucose deprivation, AMPK

## Abstract

Glucose deprivation in the slime mold *Physarum polycephalum* leads to a specific morphotype, a highly motile mesoplasmodium. We investigated the ultrastructure of both mesoplasmodia and non-starved plasmodia and found significantly increased numbers of mitochondria in glucose-deprived mesoplasmodia. The volume of individual mitochondria was the same in both growth forms. We conjecture that the number of mitochondria correlates with the metabolic state of the cell: When glucose is absent, the slime mold is forced to switch to different metabolic pathways, which occur inside mitochondria. Furthermore, a catabolic cue (such as AMP-activated protein kinase (AMPK)) could stimulate mitochondrial biogenesis.

**Electronic supplementary material:**

The online version of this article (10.1007/s00709-019-01410-1) contains supplementary material, which is available to authorized users.

## Introduction

The giant unicellular slime mold *Physarum polycephalum* grows into transport networks (termed macroplasmodia) which can reach sizes of up to square meters (Stockem and Brix 1994). Under certain nutritional conditions, i.e., a lack of glucose in the solid agar medium, and when the culture has reached a certain age, a special foraging pattern can be observed. Instead of forming a coherent network from isolated fragments, the slime mold aggregates into independent, unconnected units (termed mesoplasmodia), which then move in a straight trajectory away from their point of origin (Lee et al. 2018). Mesoplasmodia are well suited as models to study the mechanism of the slime mold’s locomotion (Oettmeier and Döbereiner 2019), because they move for hours in straight trajectories and keep a constant shape. The movement of those autonomous foraging units is comparatively fast, reaching speeds of up to 17 μm/min.

Slime molds exhibit characteristic continuous rhythmic oscillations, orchestrated by the cytoskeletal proteins actin and myosin. A detailed description of the ultrastructure can be found in Oettmeier et al. (2018). These vigorous and perpetual contractions, which are the basis for locomotion in *P. polycephalum* (Oettmeier and Döbereiner 2019), require a lot of energy in the form of ATP, which is supplied by glycolysis and oxidative phosphorylation in mitochondria. Glycolysis takes place in the cytoplasm and seems to be intensely operated by the slime mold (Sauer 1982).

**Fig. 1:**
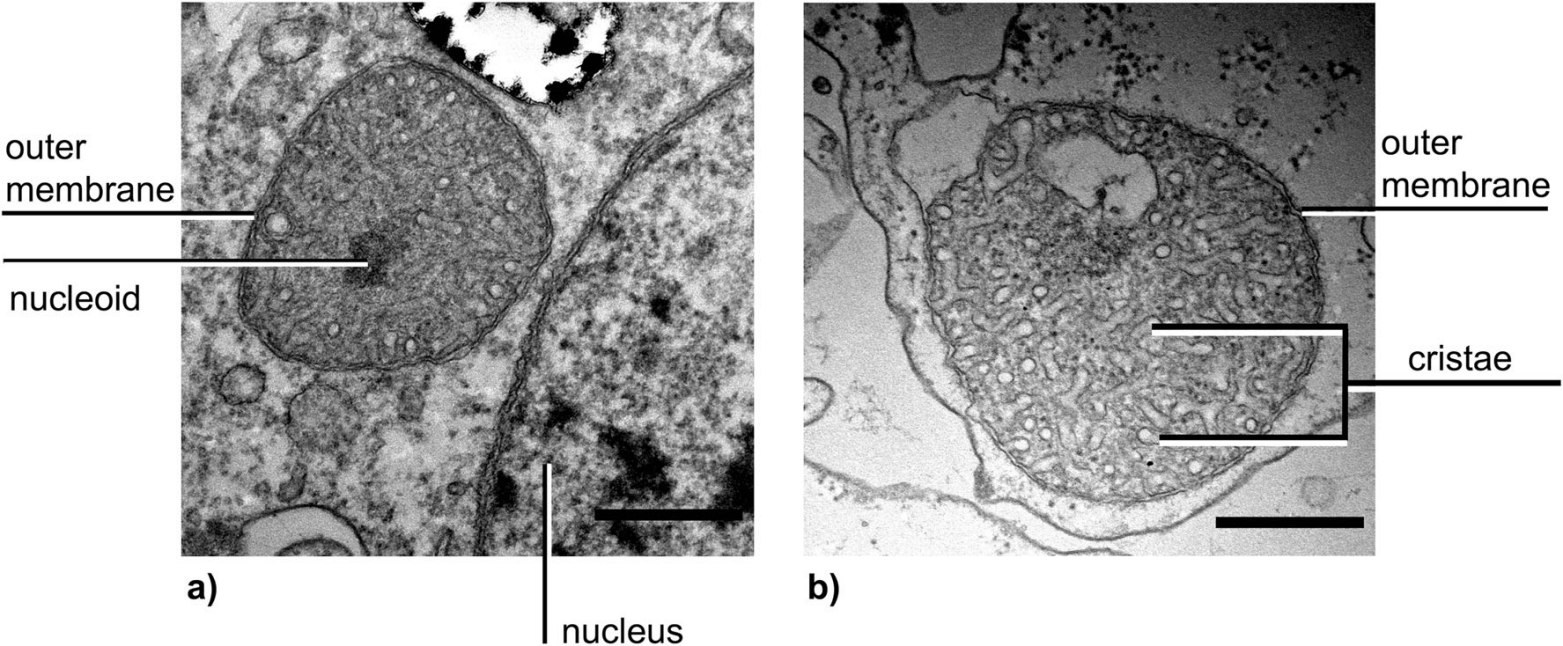
**a** Mitochondrium of a starved mesoplasmodium. **b** Mitochondrium within an unstarved plasmodium of *P. polycephalum*. Scale bars = 0.5 μm

*P. polycephalum* possesses mitochondria with tubular cristae. The ultrastructure of eumycetozoan mitochondria is unique and characteristic for slime molds (Dykstra 1977), but their function is the same as in any other eukaryotic organism. Mitochondria in *P. polycephalum* are isolated and spherical or lenticular (see Fig. 1).

Our observations of the shape conform to earlier findings (Daniel and Järlfors 1972; Sauer 1982). Mitochondria do not form networks, because the vigorous intracellular flow within the amoeboid cell body is constantly moving them around. A video of this can be found in the supplementary material (Video S1a and S1b). An elaborate mitochondrial network (as for example in budding yeast) would not be feasible due to the dynamic nature of the cytoplasm. Furthermore, it has been demonstrated that the slime mold’s mitochondria can migrate within the cell (Kuroiwa and Takahashi 1978) in response to culture conditions: they moved towards the periphery of the cell when a liquid shaking culture was left unstirred for a few hours. This migration is reversible; when the microplasmodia were agitated, they dispersed evenly again. Apart from the characteristic tubular cristae, the mitochondria possess mitochondrial DNA (mtDNA), which is packaged into the electron-dense mitochondrial nucleoid (see Fig. 1a), along with many proteins (Itoh et al. 2011). The complete mitochondrial genome has been sequenced (Takano et al. 2001).

Mitochondria perform many important biological functions. Most important is the production of ATP through oxidative phosphorylation, but they also play a role in the pronounced and well-described oscillations of the slime mold: mitochondria store and release calcium (Nations et al. 1989; Achenbach et al. 1984), thereby forming a crucial component of the biochemical oscillator. The nature and localization of this pacemaker of the contraction-relaxation cycle poses one of the most interesting problems regarding the dynamic processes of the non-muscle contractile system in *P. polycephalum*. Although the exact mechanism remains unknown, it is clear that mitochondria and the processes taking place within them are integral parts (Satoh et al. 1982; Korohoda et al. 1983). Inhibiting glycolysis or respiration leads to changes in the pattern and frequency of the oscillations.

When sufficient glucose is present in the medium, glycolysis takes place in the cytosol, producing two molecules of ATP and two molecules of NADH per molecule of glucose. Furthermore, it produces two molecules of pyruvate which are then transported into the mitochondria to enter the citric acid cycle. Electrons from the glycolysis and citric acid cycle are then being transferred by NADH and FADH_2_ to the electron transport chain, ultimately driving oxidative phosphorylation and producing more ATP (more than 30 molecules per molecule of glucose). Citric acid cycle and oxidative phosphorylation take place across the inner membrane and cristae of the mitochondria. When glucose is absent from the medium, *P. polycephalum* immediately starts to use its abundant stores of glycogen in a process termed glycogenolysis (Nader and Becker 1983). The glycogen polymer is broken down by the enzyme glycogen phosphorylase, releasing glucose, which can then be used in glycolysis. Besides glucose (and other carbohydrates), the slime mold is also able to catabolize proteins (Goodman and Beck 1974) and lipids (Poulos and Thompson 1971). If given a choice, *P. polycephalum* seems to prefer a diet that contains equal ratios of proteins and carbohydrates, or a ratio of two times more proteins than carbohydrates (Dussutour et al. 2010).

Usually, when microplasmodia of *P. polycephalum* are cultivated in a growth medium that lacks nutrients (but contains salts to maintain pH), inactive cyst-like stages are formed after a certain time (Hüttermann 1973). These spherules also occur when a shaking culture of microplasmodia depletes its liquid medium of nutrients. Depending on the starting conditions (temperature, culture volume, nutrient concentrations, shaking speed), glucose is depleted after 2.5 (Nader and Becker 1983) to 4 days (Lee et al. 2018). In this study, we used the same conditions as described in Lee et al. (2018), which means that microplasmodia reach their maximum biomass after 3 to 4 days and turn into spherules after ~ 7 days after inoculation, if left in their shaking culture. However, when microplasmodia from a 6-day-old culture are plated onto an agar plate lacking glucose, they will form the aforementioned mesoplasmodia which then begin to move outward from the inoculation center. For about 9 h, the mesoplasmodia migrate on straight trajectories without much changing their shape or showing an increase in biomass. Our samples were taken from mesoplasmodia in the middle of the migration period. After this motile phase, at around 10 h after initial plating, the mesoplasmodia reach a pause state in which migration is ceased. After this pause, mesoplasmodia either transition into static networks, continue to migrate, or move in a different pattern.

As observed in skeletal muscle cells, an elevated energy demand (e.g., through exercise) increases mitochondrial volume density (Lundby and Jacobs 2016). Similarly, myocardial hypertrophy (Wiesner et al. 1994) changes in neuronal activity (Liu and Wong-Riley 1995), and other metabolic challenges lead to an increase in mitochondrial biogenesis. Therefore, many cells are capable of adjusting their mitochondria to a change of energy demand, requiring that they possess an appropriate intracellular energy sensor. Since mitochondria are so important for both energy metabolism and the primary oscillator, we compared, in the present study, the mitochondria of glucose-deprived mesoplasmodia and non-starved plasmodia. We found a significantly increased number of mitochondria in glucose-deprived mesoplasmodia. We hypothesize that mitochondrial biogenesis is stimulated in *P. polycephalum* mesoplasmodia grown in the absence of glucose, probably in order to compensate for the reduced supply of glycolytic ATP and pyruvate.

## Material and methods

### Microplasmodia culture

We used the strain WT33 (Marwan and Starostzik 2002) × LU898 (Kawano et al. 1987), which was kindly provided by Prof. Dr. Wolfgang Marwan (Universität Magdeburg). Microplasmodia were grown in a liquid growth medium (see Tables 1 and 2). The cultures were grown at a constant temperature of 24 °C and rotation speed (180 rpm) in the dark. Torn apart by shear forces, multiple small and spherical units are produced, whose size is determined by the shaking speed. Fresh microplasmodia cultures were prepared by taking 2 ml of the previous culture at days 3 to 4, centrifuging gently and discarding the supernatant. The pellet was then transferred into new liquid medium.

**Table 1:** Liquid growth medium for microplasmodia

Ingredient	Amount (for 1 l)
Bacto tryptone	10 g
Yeast extract	1.5 g
D(+) glucose monohydrate	11 g
Anhydrous citric acid	3.54 g
Iron(II)sulfate heptahydrate	0.084 g
Calcium chloride dihydrate	0.6 g
Potassium dihydrogen phosphate	2 g
100 × MMZ solution	10 ml
Fill up to 1 l with MilliQ water	
pH adjusted to 4.6 with 4 N NaOH

**Table 2:** MMZ solution

Ingredient	Amount (for 1 l)
Magnesium sulfate heptahydrate	60 g
Manganese(II)chloride dihydrate	6 g
Zinc sulfate heptahydrate	3.4 g

### Mesoplasmodia

To create mesoplasmodia, microplasmodia from a 6-day-old liquid culture were centrifuged, the supernatant discarded, and they were resuspended briefly with MilliQ water. Microplasmodia were then transferred onto a semi-defined medium (SDM) agar plate lacking glucose (see Table 3).

**Table 3:** 2 × SDM agar without glucose

Ingredient	Amount (for 1 l)
Bacto soytone	20 g
Anhydrous citric acid	7.08 g
Iron(II)chloride tetrahydrate	0.078 g
D(+) biotin	0.01 g
Thiamin hydrochloride	0.08 g
Potassium dihydrogen phosphate solution, 80 g l^−1^	50 ml
Calcium chloride dihydrate solution, 41.2 g l^−1^	50 ml
Magnesium sulfate heptahydrate solution, 24 g l^−1^	50 ml
EDTA disodium salt dihydrate solution, 9.2 g l^−1^	50 ml
Zinc sulfate heptahydrate solution, 136 g l^−1^	0.5 ml
Fill up to 1 l with MilliQ water	
pH adjusted to 4.6 with 4 N NaOH	
Agar	17 g
Dissolve in MilliQ water, then autoclave	500 ml
Add 2 × SDM medium	500 ml
Add hemin solution, 0.5 g l^−1^	10 ml
Pour into Petri dishes	

*P. polycephalum* requires hemin to grow (Daniel et al. 1962). However, hemin is poorly soluble in water. Therefore, it needs to be dissolved in 1 N NaOH first. This solution can then be mixed with MilliQ water to achieve the desired final concentration.

SDM agar contains an additional 20 g of D(+) glucose per liter when used to grow typical macroplasmodial networks. On the glucose-deficient agar, the microplasmodia form aggregates and fuse with each other. After about 3 h, the first migrating units leave the initial patch and move radially outwards.

### Transmission electron microscopy

Macroplasmodia and mesoplasmodia growing on an agar surface were submerged with a fixative (80 mM KCl, 50 mM sodium cacodylate, pH 7.2, 20 mM NaCl, 2.5% glutaraldehyde). The fixation was carried out for at least 30 min on ice. Plasmodia were fixed together with the agar they were growing on, which was then cut into blocks the size of a few square millimeters with a scalpel. Post-fixation and contrast enhancement was carried out in 2% OsO_4_ on ice for 60 min. After post-fixation, the samples were washed thoroughly with double-distilled water. Samples were then left to contrast for 12 h at 4 °C in the dark in 0.5% uranyl acetate. Uranyl acetate helps to increase the contrast as well as the stability of the fine structures of the cell. After fixing and contrasting, the samples were dehydrated by passing them through a series of increasing ethanol concentrations. First, they were treated with 30% ethanol for 30 min, whereby the ethanol was exchanged after 15 min. This procedure was repeated for 50%, 70%, 90%, and 100% ethanol. An extra step was performed with 100% ethanol, dehydrated with a molecular sieve (15 min).

Next, the specimens were embedded in resin. Embedding was performed with glycid ether 100 (Luft 1961). The ethanol was replaced in a descending alcohol series with the glycid ether 100. The glycid ether solutions A and B were mixed at a ratio 3:7 (A:B). A descending alcohol series was prepared as follows at room temperature: First, the samples were infiltrated for 15 min with a mixture of 100% ethanol and glycid ether A + B at a ratio of 3:1, then at a 1:1 ratio for 15 min, and finally at a 1:3 ratio for 15 min. The ethanol-resin mixture was carefully removed and the last step was infiltration of the tissue with a pure glycid ether (A + B) mixture for a total of 45 min, whereby the glycid ether was replaced twice after 15 min each. After the glycid ether was removed for the last time, the accelerator DMP-30 was added to the mixture. The samples were then left to polymerize in a vacuum oven at 60 °C for 2 to 3 days. Ultra-thin cutting (40–60 nm) was performed with a Reichert-Jung Ultracut E microtome. TEM was carried out on a Zeiss EM 900, equipped with a water-cooled frame-transfer-CCD-camera (TRS). Images were acquired using a PC with the software “ImageSP” (TRS).

### Stereological measurements

Stereology is defined as a set of mathematical methods which relate parameters defining three-dimensional structures to measurements obtained from two-dimensional sections. In other words, one can estimate higher dimensional information from lower dimensional samples. The advantage of evaluating thin sections with stereology is that it yields quantitative morphological data. Geometric properties of structures (e.g., mitochondria) embedded in a referent space (e.g., cytoplasm) can be estimated by studying the intersection of these structures with a probe. Probes are points, lines, or grids which are being superimposed onto the section (image). A prerequisite for stereology is that samples must be uniform, isotropic, and random (UIR); this means that the orientation of the cut surface and the position of the embedded specimen must be random, as well as the positioning of probes. We use the following stereological relationships:

#### Volume density

Volume density (*V*_V_) or volume fraction is the ratio between the volume of the structure and the volume of the referent space (Eq. 1).

1 $$ {V}_{\mathrm{V}}=\frac{\Sigma {P}_{\mathrm{i}}}{\varSigma {Q}_{\mathrm{i}}\ } $$

**Fig. 2:**
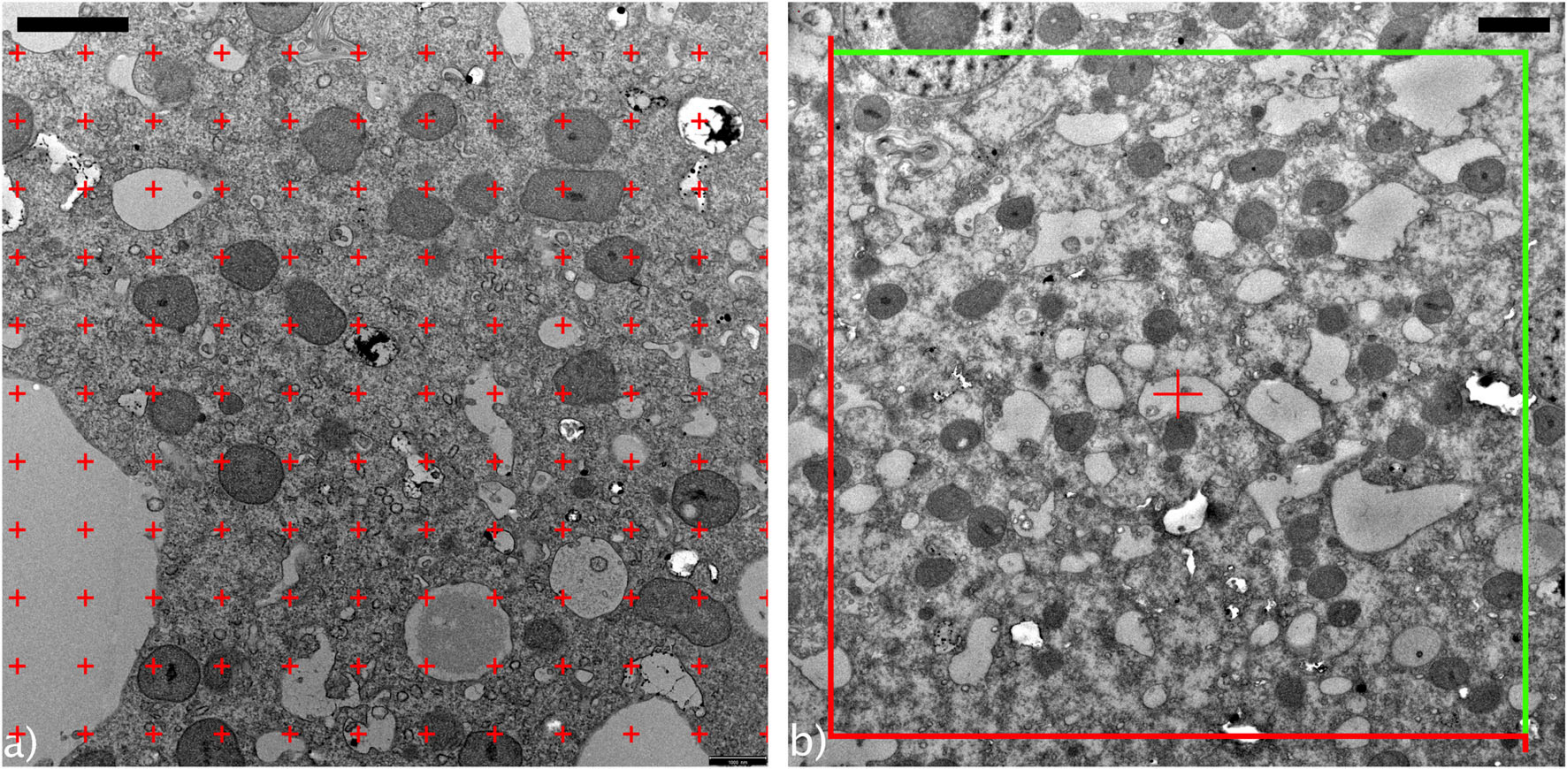
**a** Random offset grid. The number of points (red crosses) which fall on mitochondria is counted (*P*_i_), as well as the number of points which falls onto the referent volume (cytoplasm, *Q*_i_). This takes into account the relatively high porosity of the slime mold’s cytoplasm. Scale bar = 2 μm. **b** Counting frame. Green line = inclusion line, red line = exclusion line. Scale bar = 2 μm

The probes used are points. A point grid is superimposed onto the TEM image and mitochondria are counted which coincide with the probes (see Fig. 2a).

By doing a point count on an image (*i*), we obtain the number of points which fall onto mitochondria (*P*_i_) and the number of points in the referent space (i.e., cytoplasm, *Q*_i_). The volume density states which percentage of the cytoplasm is occupied by mitochondria. FIJI provides grids with random offset. Depending on magnification, the area per point was chosen to lie between 1 and 5 μm^2^ (110–361 points per image).

#### Numerical density

Numerical density (*N*_V_) is the number of structures per volume of the referent space (i.e., number of mitochondria per unit volume of cytoplasm). In this case, the probes are volumes. Since the mitochondria of *P. polycephalum* are ellipsoid in shape and of similar sizes, we can use the method proposed by Weibel and Gomez (1962). First, using a counting frame (a macro (Mironov 2014) implemented in FIJI), the number of structures per unit area (*N*_A_) is computed (see Fig. 2b). Following stereological rules, a mitochondrion is only counted if it lies entirely within the counting frame or if it touches a green inclusion line. It is not counted if it intersects a red exclusion line. Second, we need to calculate *ϵ*, i.e., the ratio of short (*a*) to long semi-axis (*b*) (Eq. 2).

2 $$ \epsilon =\frac{a}{b} $$

Here, *ϵ* is approximately 0.86, indicating a slightly prolate ellipsoid (*ϵ* < 1). For each *ϵ*, the corresponding shape factor *β* has to be obtained from literature (Weibel and Gomez 1962). In our case, *β* is 1.4. For a perfect sphere, *ϵ* = 1 and *β* = 1.38. The numerical density (*N*_V_) can now be calculated using Eq. 3.

3 $$ {N}_{\mathrm{V}}=\left(\frac{1}{\beta}\right)\frac{N_A^{\frac{3}{4}}}{V_V^{\frac{1}{2}}} $$

#### Mean mitochondrial volume

The mean volume $\overline{V}$ of mitochondria can be calculated from *V*_V_ and *N*_V_ using Eq. 4 (Cruz-Orive and Weibel 1990):

4 $$ \overline{V}=\frac{V_{\mathrm{V}}}{N_{\mathrm{V}}} $$

### Autofluorescence

Autofluorescence imaging of microplasmodia was performed using a Zeiss Axio Oberver.Z1 equipped with a Zeiss incubation system consisting of Heating Unit XL S and Temp Module S. Imaging was carried out at 24 °C. A Zeiss Plan Apochromat × 40 with a numerical aperture of 0.95 was used, and images were taken by a Zeiss Axio-Cam MRm. Microplasmodia were plated onto Petri dishes with thin glass bottoms. They were illuminated at a wavelength of 380 nm with a Zeiss HXP 120 mercury lamp, of which the UV filter was removed. We used a 79000 ET FURA 2 Hybrid filter set (Chroma) and a Zeiss 76 HE reflector filter set.

## Results

### Volume fraction, number density, and mean volume

**Fig. 3:**
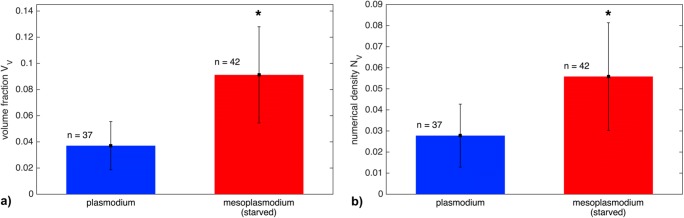
**a** Volume fraction (*V*_V_) of mitochondria in unstarved (blue) and starved (red) slime mold. Starved mesoplasmodia contain a significantly higher volume fraction of mitochondria (*p* < 0.001) than unstarved specimens. **b** Numerical density (*N*_V_) [μm^−3^] of mitochondria in unstarved and starved slime mold. Again, the difference is statistically significant (*p* < 0.001). Error bars = standard deviation *σ*

We compared TEM images of three specimens each of glucose-deprived mesoplasmodia and non-starved plasmodia. On average, mitochondria in unstarved plasmodia occupy ~ 4% of the cytoplasm volume, but ~ 9% in starved mesoplasmodia (Fig. 3).

**Fig. 4:**
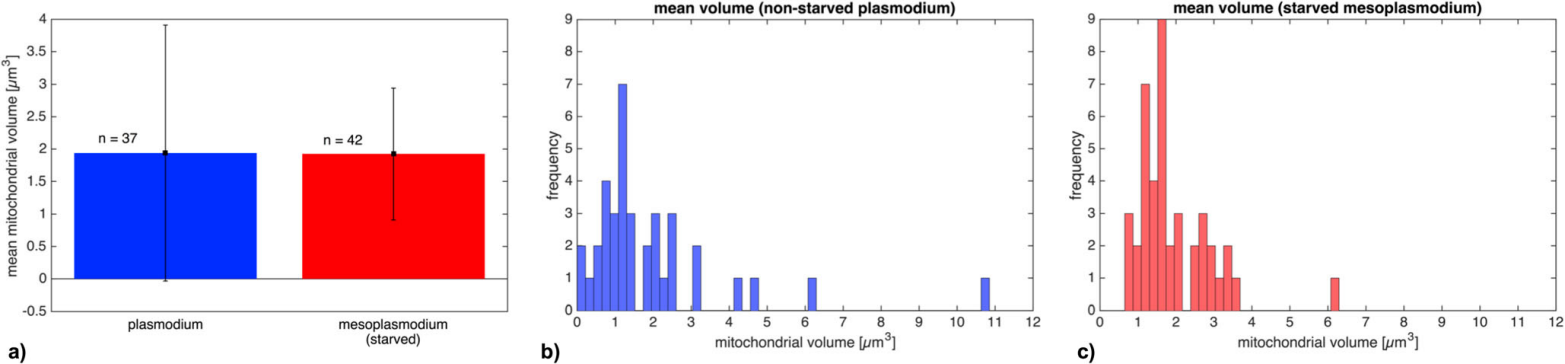
**a** Mean mitochondrial volume of unstarved (blue) and starved (red) slime mold. Error bars = standard deviation *σ*. **b** Histogram of mitochondrial volume (unstarved plasmodium) **c** Histogram of mitochondrial volume (starved mesoplasmodium)

The results for the numerical density (*V*_V_) are similar: Unstarved plasmodia contain ~ 0.035 mitochondria per μm^−3^, whereas starved mesoplasmodia contain ~ 0.08 mitochondria per μm^−3^. There are approximately twice as many mitochondria per unit volume of cytoplasm in mesoplasmodia than in unstarved plasmodia. Both the results for *V*_V_ and *N*_V_ show statistically highly significant differences as confirmed by two-sample *T* tests (both *p* < 0.001). The mean mitochondrial volume, however, does not vary between starved and unstarved plasmodia (see Fig. 4).

### Autofluorescence

**Fig. 5:**
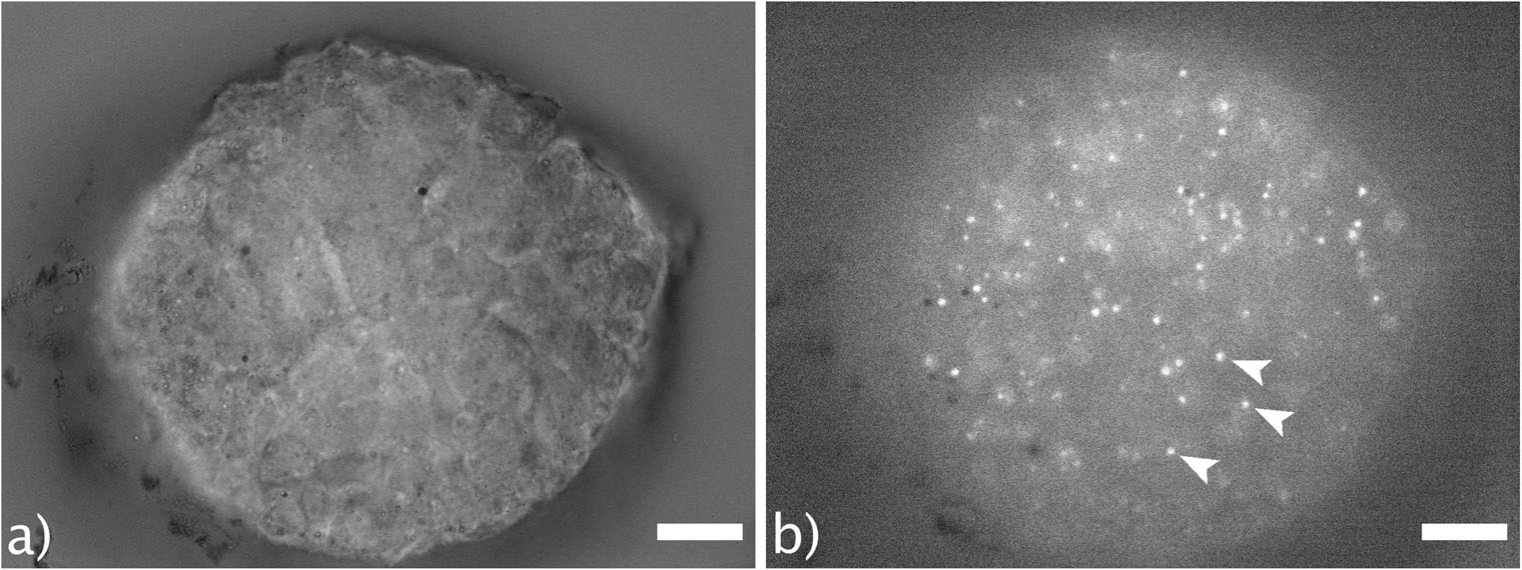
**a** Bright field image of a microplasmodium from a shaking culture. **b** Fluorescence image. The same microplasmodium was illuminated with 380-nm wavelength light. Arrow heads point to mitochondria. Scale bars = 25 μm

When living plasmodia are illuminated with wavelengths in the range of 340 to 380 nm, they show pronounced autofluorescence with an emission wavelength of around 460 nm. A base autofluorescence is detectable in the cytoplasm, as well as brightly fluorescing spots (see Fig. 5b).

We deduce that these spots are mitochondria. This conclusion is based on the presence of NAD and its reduced form, NADH, in both mitochondria and cytoplasm. NADH strongly absorbs ultraviolet light, with an emission peak at 460 nm. In small amounts, NADH is produced during glycolysis, which explains the low fluorescence of the cytoplasm. However, the largest share of the cell’s NADH is found inside the mitochondria (Ince et al. 1992), accounting for the strong autofluorescence. The autofluorescence data also confirms our stereological finding that mitochondria do not form networks, but are rather isolated organelles (see Video S1b). NADH autofluorescence can be used to assess intracellular pH (Ogikubo et al. 2011), monitor mitochondrial toxicity (Rodrigues et al. 2011), and can generally give insight into the energy metabolism (Bartolomé and Abramov 2015; Evans et al. 2005; Mayevsky and Rogatsky 2007). However, in *P. polycephalum*, this autofluorescence can cause problems when short-wavelength calcium-staining dyes, such as Fura 2, are used. A video of a living microplasmodium exhibiting autofluorescence (Video S1b) and the same microplasmodium at bright field illumination (Video S1a) can be found in the supplementary material.

## Discussion

Our results show that under glucose-deprived conditions, the number of mitochondria is significantly increased. Their volume does not differ between glucose-deprived and unstarved plasmodia, indicating that this is not an instance of fragmentation. In contrast, in mouse embryonic fibroblasts, glucose depletion leads to increased mitochondrial fragmentation (Rambold et al. 2011). Likewise, in yeast, glucose deprivation under aerobic conditions leads to a fragmentation of mitochondria into many small units (Visser et al. 1995). However, how cells respond in detail to glucose withdrawal is not well studied, and results are controversial (Song and Hwang 2019; Wappler et al. 2013). In cancer cells, for example, glucose deprivation causes cell death (Iurlaro et al. 2017). However, the ability to reprogram the energy metabolism is a hallmark of cancer (Hanahan and Weinberg 2011). In other cell types, viability is not significantly affected (Jelluma et al. 2006). It seems that there is a great variability in the response to glucose depletion, depending also on cofactors like a simultaneous lack of oxygen. For example, after a non-lethal phase of both oxygen and glucose depletions, an increase in mitochondrial biogenesis in neurons was observed (Wappler et al. 2013).

Apart from fragmentation, mitochondrial morphology can be affected by the metabolic state of the cell. Starved amoeba of the species *Chaos carolinense* exhibited highly organized special membrane structures within their mitochondria (Chong et al. 2018). During starvation-induced autophagy, mitochondria can increase in size and become elongated in shape, which optimizes ATP production and spares them from being digested (Blackstone and Chang 2011). In other words, stress can affect mitochondrial morphology. Our results show no difference in morphology between glucose-deprived and non-starved plasmodia. A difference to the studies cited above, however, is that apart from a lack of glucose, the medium contained a source of protein (see Table 3). Soytone is an enzymatic digest of soybean meal, and it contains peptides, amino acids, vitamins, and complex carbohydrates. Those are alternative energy sources that the slime mold can metabolize, and therefore, neither the increase in number nor the morphology is related to stress.

**Fig. 6:**
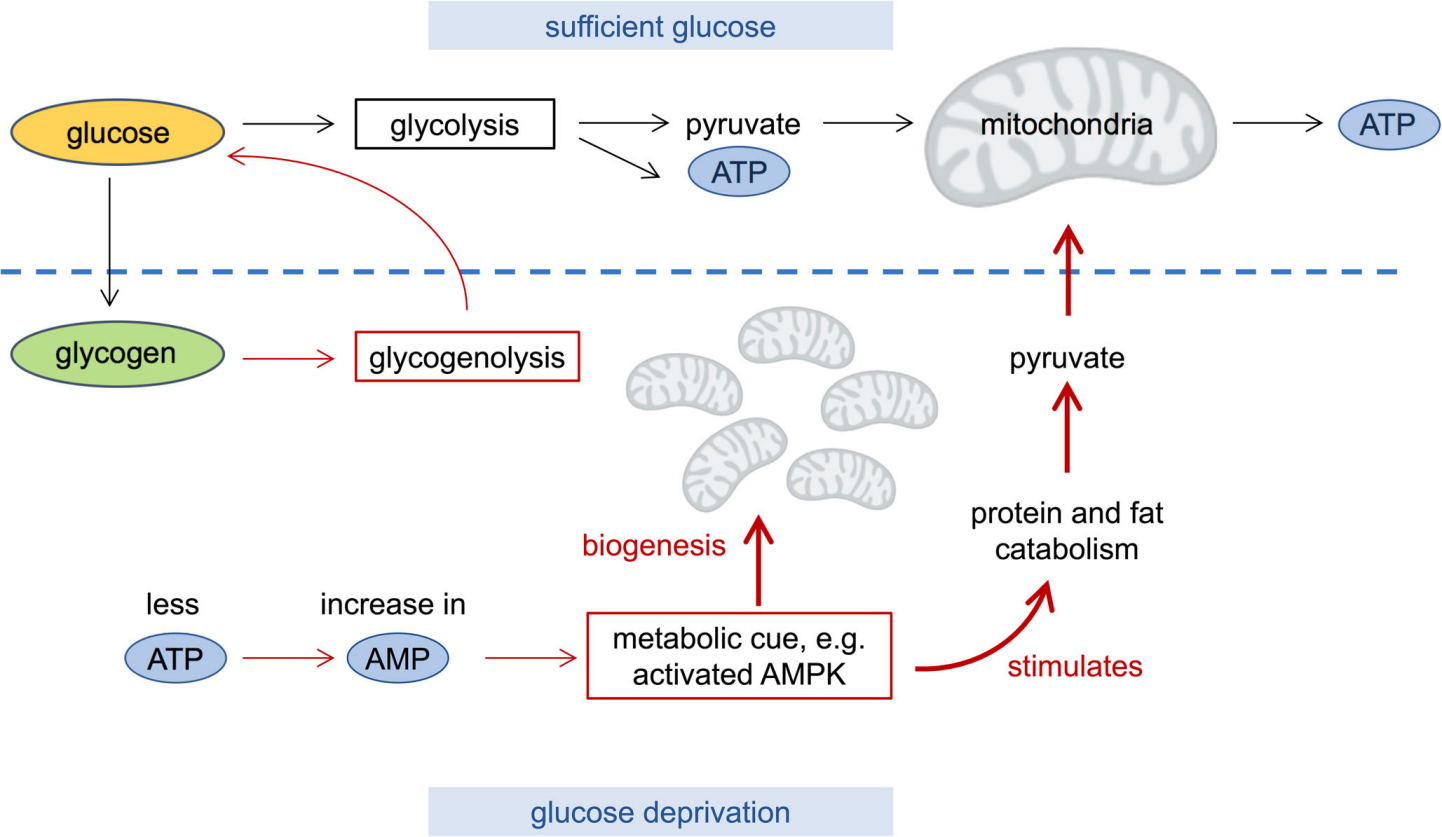
Proposed metabolic control of mitochondrial number. Explanation is given in the text

Our results show that mitochondrial biogenesis is stimulated in *P. polycephalum* grown in the absence of glucose, probably in order to compensate for the diminished supply of glycolytic ATP and pyruvate. We speculate that the number of mitochondria correlates to the metabolic state of the cell (see Fig. 6).

The increase in mitochondrial numbers leads to a higher ATP production. In conditions where glucose is abundant (upper panel in Fig. 6), glucose is converted to ATP and pyruvate via glycolysis. At the same time, the slime mold stores surplus energy in the form of glycogen (Goodman and Rusch 1969; Nader and Becker 1983). Pyruvate is then transported into the mitochondria, where it enters the citric acid cycle, and during oxidative phosphorylation, more ATP is produced. This seems to be the preferred metabolic pathway when sufficient glucose is present. However, when glucose is withdrawn (lower panel in Fig. 6), different metabolic pathways are taken. First, *P. polycephalum* uses up its glycogen storages. Glycogenolysis releases glucose from glycogen, which then enters glycolysis. Nader and Becker (1983) have measured that after the glucose in the growth medium was depleted, glycogen stores within microplasmodia lasted for a period of ~ 5.5 days until it ran out. As soon as exogenous glucose is consumed or removed, glycogen is degraded.

Another pathway during glucose depletion starts with lower levels of ATP in the cell, with a simultaneous increase in AMP. This is a metabolic cue, which leads to the activation of AMP-activated protein kinase (AMPK). This enzyme belongs to a highly conserved protein family with orthologs in yeast (*Saccharomyces cerevisiae* (SNF1)) (Hedbacker and Carlson 2008), in other fungi, in plants (SnRK1) (Margalha et al. 2016), and in *Dictyostelium discoideum* (Bokko et al. 2007), a member of the amoebozoa group of organisms to which *Physarum* also belongs. Several AMPK orthologs are encoded in the *P. polycephalum* genome (Schaap et al. 2016) and are expressed in starving, sporulation-competent plasmodia (Glöckner and Marwan 2017). AMPK plays a role in cellular energy homeostasis. When ATP levels lower, AMPK activation stimulates, among other processes, fatty acid oxidation and mitochondrial biogenesis (Mihaylova and Shaw 2011; Song and Hwang 2019). Furthermore, AMPK enhances protein catabolism (He et al. 2017). AMPK is an intracellular energy status sensor and key regulator of mitochondrial biogenesis. When activated by low ATP levels, AMPK triggers a metabolic switch, decreasing the activity of anabolic pathways and enhancing catabolic processes to restore the energy balance.

In summary, we propose that an imbalance between energy requirement and energy supply (deprivation of glucose) regulates mitochondrial biogenesis. By withdrawing glucose from the culture medium, we forced the slime mold to be exclusively dependent on mitochondrial ATP production. As a result, mitochondrial biogenesis was increased and we found a very high number of mitochondria. Additionally, mesoplasmodia are migrating fast and far in search for food, and this locomotion is also very energy consuming. To compensate for a lack of glycolytic ATP, mitochondrial numbers are increased. Our findings highlight the importance of the AMPK-like metabolic switch in *P. polycephalum*. This pathway has not yet been confirmed for glucose-deprived mesoplasmodia, but appears to be a very likely candidate to explain our observations. A closer investigation of this sophisticated system of energy metabolism adaptation is needed in order to get a more complete understanding of how *P. polycephalum* manages homeostasis in the face of nutritional challenges.

## Electronic supplementary material

Video S1 a: Bright field video of a living microplasmodium. Frame rate = 10 s. (AVI 15585 kb)

Video S1 b: Autofluorescence of the same microplasmodium. Illumination wavelength = 380 nm, frame rate = 10s. (AVI 50107 kb)
